# Sampling and processing matter in airway microbiota discovery

**DOI:** 10.1186/s13054-024-04971-7

**Published:** 2024-05-31

**Authors:** Sébastien Imbert, Mathilde Revers, Raphaël Enaud, Arthur Orieux, Laurence Delhaes, Renaud Prével

**Affiliations:** 1 Mycology-Parasitology Department, CIC 1401, CHU Bordeaux, 33000 Bordeaux, France; 2 Centre de Recherche Cardio-Thoracique de Bordeaux, Inserm UMR 1045, Univ Bordeaux, 33000 Bordeaux, France; 3 CRCM Pédiatrique, CIC 1401, CHU Bordeaux, 33000 Bordeaux, France; 4 Medical Intensive Care Unit, CHU Bordeaux, 33000 Bordeaux, France

Dear Editor,

We read with interest the comment by Wang and Li [1], related to our previous article showing differences in lower airway microbiota composition according to acute respiratory distress syndrome (ARDS) etiologies and published in Critical Care [2]. We totally agree with Wang and Li that viral-related (COVID-19 or influenza) and bacterial-related ARDS share different pathophysiological features, reinforcing the relevance to analyze these groups of patients separately, and that sample selection and processing are critical steps for lower airway microbiota studies, as well as bioinformatic analyses.

Regarding selection of patients, it is unlikely that we included patients who not only have COVID-19 or influenza infections, but also concurrent other infections, as routine bacteriological culture from endotracheal aspirate (ETA) remained sterile for these patients.

Regarding the impact of ETA versus bronchiolo-alveolar lavage (BAL) as sampling method, we do not think that the choice of BAL by an unpublished ongoing prospective, multicenter study (NCT06114784) is a scientific evidence of its superiority compared to ETA for microbiota investigation. We agree with Wang and Li that ETA is more often contaminated by upper tract microorganisms than BAL, nevertheless BAL is performed at the 4th bronchial division whereas alveoli stand at the 23rd and it can also be contaminated. No data stand regarding the comparison between ETA and BAL for microbiota investigation but in ventilator-associated pneumonia, microbial over-identification of ETA culture was 16% using BAL culture as the reference standard [3], confirming that ETA is not that over-contaminated compared to BAL. In addition, BAL is known to represent only the washed region of lungs (exhibiting a wide variability in microbiological results by sampling different lung sites, even comparing left and right lungs [4]), while ETA represents a more global sampling of both lungs. For these reasons, we believe that no firm conclusion can be drawn regarding the superiority of ETA or BAL for microbiota investigation. To our opinion, it is the homogeneity of the sampling method across the different groups which is of crucial importance, which was formally completed in our study.

As the samples included in our study were collected during two periods (one pre-COVID-19 pandemic and one during the early stages of the COVID-19 pandemic), we first assessed the impact of sample processing to avoid bias in our analyses. For this purpose, we compared the bacterial and fungal microbiota composition among 14 respiratory samples (10 COVID-19 positive and 4 negative) processed by our standard extraction protocol [2] and by a protocol with prior chemical and heat lysis sample, as used in routine in the early COVID-19 pandemic. As shown in Fig. 1, we did not observe any significant differences in alpha-diversity for both V3-V4 regions of 16S rRNA (Fig. 1A) and ITS2 (Fig. 1B) targets between the two protocols. Moreover, Bray–Curtis analysis of ß-diversity showed that the microbiota composition was not dissimilar between the two protocols for both bacteriobiota (Fig. 1C) and mycobiota (Fig. 1D).

**Fig. 1:**
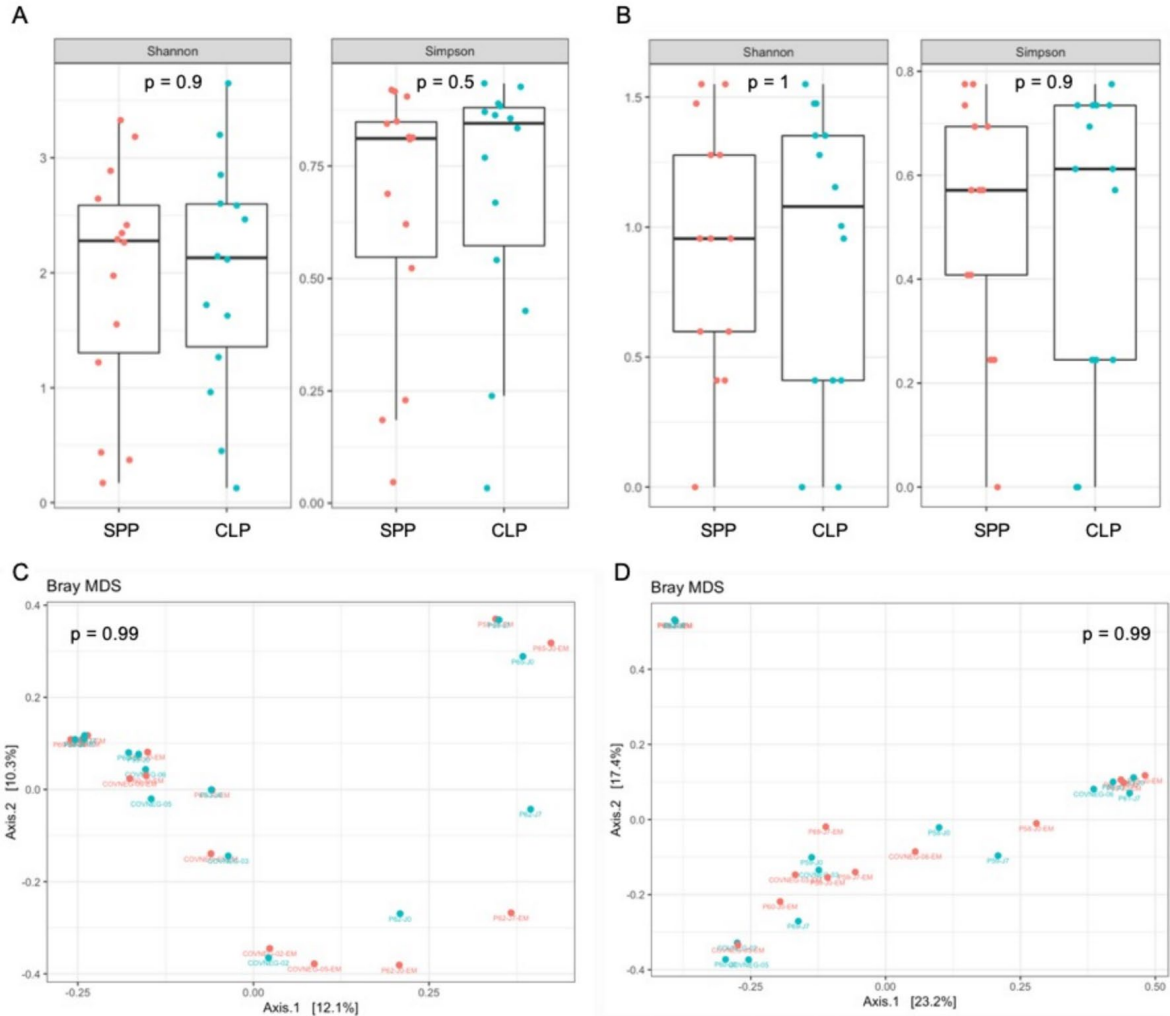
Lower airway microbiota compositions according to sample processing protocols. Boxplot of lower airway bacteriobiota (**A**) and mycobiota (**B**) α-diversity estimated by Shannon and Simpson indices according to sample processing protocols. Metric Bray–curtis analysis of β-diversity of lower airway bacteriobiota (**C**) and mycobiota (**D**) between standard processing protocol (SPP, red dots) and chemical lysis protocol (CLP, blue dots). SPP: standard processing protocol (red dots), CLP: chemical lysis protocol (blue dots)

In conclusion, these results give us strong confidence to ensure the absence of bias related to sample period and processing in our analysis and strengthen our conclusion on the impact of the ARDS etiology in lower airway microbiota composition.

## Data Availability

The 16S rRNA gene and ITS2 sequences have been submitted to the European Nucleotide Archive (Accession N° ERP134910). The scripts used for bioinformatics analysis during the current study are available in the Supplemental Materials of a previous study from our team (10.1128/spectrum.05062-22).
